# In vitro effects of potato glycoalkaloids on plant-pathogens, beneficial microbes, and *Arabidopsis thaliana*

**DOI:** 10.1038/s41598-025-19637-9

**Published:** 2025-09-17

**Authors:** Marília Bueno da Silva, Franziska Genzel, Anika Wiese-Klinkenberg, Sandra Bredenbruch, Florian M. W. Grundler, A. Sylvia S. Schleker

**Affiliations:** 1 Institute of Bio- and Geosciences (IBG-4: Bioinformatics), CEPLAS, Forschungszentrum Jülich GmbH, 52425 Jülich, Germany; 2 Bioeconomy Science Center, BioSC, Forschungszentrum Jülich GmbH, 52425 Jülich, Germany; 3 Institute of Crop Science and Resource Conservation (INRES: Molecular Phytomedicine), University of Bonn, 53115 Bonn, Germany

**Keywords:** Glycoalkaloids, Bioactivity, Growth reduction, Natural compounds, α-chaconine, Plant pathogen, Biotechnology, Microbiology, Plant sciences

## Abstract

Potato glycoalkaloids (PGAs), α-solanine and α-chaconine, are secondary metabolites related to plant defense. Highly concentrated in the upper part of potato plants, they exhibit antimicrobial properties. Seeking more sustainable crop protection strategies, this study investigates the effects of PGAs on plant pathogens and beneficial organisms. These organisms were exposed to different PGA concentrations (0.98 to 250 ppm), with evaluations focusing on developmental and survival metrics. Key findings highlight α-chaconine as the more potent compound, causing significantly stronger adverse effects across tested organisms. E.g., α-chaconine (≤ 25 ppm) reduced nematode mobility by 43%, host attraction by > 45%, and infection rates by 63%. At 250 ppm, α-solanine moderately reduced mycelial growth, while α-chaconine reduced it by 78%. Crucially, beneficial organisms experienced minimal growth impairment (≤ 19%) even at the highest concentration (250 ppm). *Arabidopsis thaliana* seedling development was impaired by both PGAs, and seedlings exposed to these compounds exhibited a strong, transient oxidative burst, indicating direct stress activation. Pretreatment with PGAs did not induce priming effects but even decreased subsequent elicitor-induced responses. The selective toxicity against pathogens, coupled with minimal impact on beneficial species, positions PGAs, particularly α-chaconine, as promising starting points for sustainable crop protection strategies.

**Supplementary Information:**

The online version contains supplementary material available at 10.1038/s41598-025-19637-9.

## Introduction

Glycoalkaloids are secondary metabolites predominantly found in plants from the *Solanum* genus, including crop plants like tomato (*Solanum lycopersicum* L.), potato (*S. tuberosum* L.), and eggplant (*S. melongena* L.)^1^. Other non-crop plants rich in glycoalkaloids are e.g., *S. nigrum* L. and *S. pseudocapsicum*, both of which are well-known for their use in traditional medicine^2,3^. The two best-known glycoalkaloids in potatoes are solanine and chaconine, which are present in all tissues at varying concentrations and ratios^4^.

Previous studies^5,6^ reported that concentrations of solanine and chaconine are highest in potato sprouts, followed by flowers, leaves, and berries (non-edible). Due to their high toxicity to humans, potato cultivars have been bred to reduce glycoalkaloid concentrations in tuber flesh^7,8^. Although the potato waste industry focuses mainly on pulp and peel for extraction of bioactive molecules^9 ^the high concentration of glycoalkaloids in non-edible parts of potato presents itself as a good opportunity to increase the profits from potato cultivation. This action could improve the circular bioeconomy in potato fields^4^.

Chemically, glycoalkaloids are steroidal glycosides that consist of a C27-skeleton aglycone called solanidine, attached at the 3-OH position to a carbohydrate side chain of trisaccharides^10^. Solanine and chaconine are predominantly present (95%) as α-forms; the remaining 5% consist of β- and γ-forms, which are produced through the hydrolysis of α-form molecules^1,11^. The most pronounced difference between the two glycoalkaloids is their carbohydrate side chain composition. Solanine contains a solatriose chain comprising one glucose, one galactose, and one rhamnose molecule, whereas α-chaconine contains a chacotriose chain composed of one glucose and two rhamnose molecules^12^. Numerous studies^13–15^ have reported that a difference in sugar moieties contributes to distinct biological activities. According to Rayburn et al.^12^, toxicity levels may rely on the sugar moiety’s type, number, and stereochemical orientation. A commonly cited mode of action for potato glycoalkaloids is membrane disruption. Since glycoalkaloids molecules have an affinity for membrane sterols, they can form a rigid matrix resulting in lytic activity^16^.

As secondary metabolites, potato glycoalkaloids (PGAs) are usually related to plant defense against pests and pathogens and might be useful as pesticides applied to plants^17^. Also, content and composition of glycoalkaloids might serve as a trait for breeding resistant potato cultivars^18,19^. However, the contribution of PGAs to pathogen resistance/tolerance is context-dependent and needs to be carefully assessed. When it comes to cultivated potato (*S. tuberosum*), some studies showed that the presence of glycoalkaloids could influence fungal and nematode infection. For instance, Fewell and Roddick^20^ demonstrated that α-chaconine impaired *Alternaria brassicicola* and *Phoma medicaginis*’ morphology by inhibiting spore germination, reducing hyphal thickness, and decreasing branching. Udalova et al.^21^ also observed the effect of externally applied α-chaconine (spray application) in decreasing the number of knots, female size, and number of eggs from the root-knot nematode *Meloidogyne incognita*, on tomato plants.

In contrast, no evidence was found by Desmedt et al.^22^ to conclude that the basal content of PGAs in potato plants correlates with pathogen resistance to the cyst nematode *Globodera pallida* in the wild potato specie *Solanum canasense* or in a potato breeding population with varying content of PGAs. However, the ability to induce PGAs in case of infection correlated well with resistance against the oomycyte *P. infestans* in tubers of resistant cultivars^19^. Baur et al.^19^ discuss the importance of local infection induced accumulation at infection sites being more relevant than basal concentrations.

Research on wild relatives of cultivated potato and their different glycoalkaloids, revealed a variation in effect among both the aglycone and the sugar moiety of the glycoalkaloids. A stronger inhibitory effect was recorded when tetraose glycoalkaloids were compared to triose glycoalkaloids in *Solanum chacoense*. Tetraose GAs such as commersonine, leptine, dihydrosolanine, and dihydrochaconine promote a stronger effect than the triose GAs α-solanine and α-chaconine on reducing foliar defoliation by Colorado Potato Beetle (*Leptinotarsa decemlineata*) (CPB)^23^. Wolters et al.^18^ recently detected tetraoses (here (dehydro-) commersonine and demissine) in *Solanum commersonii* plants resistant to CPB and *Alternaria solani*. Trioses, on the other hand, were present only in susceptible plants. Similar outcomes in relation to tetraoses x CPB-resistant species were also recorded by Tai et al.^24^.

In order for crops to thrive and have product quality in crop fields, they must be in good state of health. Constantly, this health is threatened by pests and pathogens, including insects, weeds, fungi, viruses, nematodes, and others^25,26^. For example, plant-parasitic nematodes (PPNs) are a group of pathogens that cause substantial crop losses by colonizing roots, which leads to reduced water and nutrient uptake^27,28^. Therefore, identifying effective alternatives for pest and pathogen control is essential. Integrated management strategies typically combine sustainable approaches such as the use of resistant varieties, biological agents, synthetic nematicides, and crop rotation^28^.

Natural compounds derived from biological systems, such as microbes, animals, or plants, are a compatible alternative to integrated in management strategies^29^. Compared to synthetic chemicals, many of these natural compounds are safer and less harmful to non-target organisms^30,31^ but this has to be investigated carefully in each case. Some natural substances, such as pyrethrins, spinosad, sulfur, and even hydrogen peroxide, carry toxicity risks to aquatic and human life upon ingestion or inhalation^32^. Other examples previously recorded are: Rotenone, a bioinsecticide extracted from *Derris* and *Lonchocarpus* vegetables which contains the isoflavonoid rotenone in its composition, showed high toxicity against fish and insects due to its rapid uptake and inhibition of respiratory electron transport system I^33^; and *Tephrosia vogelii*, a plant that has been used in Africa as a pesticide and soil ameliorant, but which also contains rotenone and demonstrated to be toxic to fish^34^.

Natural compounds can present rapid biodegradation; however, this characteristic can walk in two directions: while it positively reduces their level of environment contamination^35^ their short activity and less persistence also require more frequent rounds of application to maintain efficacy^29^. When a high frequency of exposure is present in protection practices, the consequent increase in selection pressure might lead to resistance cases^36^.

Resistance to different types of natural compounds have been reviewed by Siegwart et al.^36^. Resistance can develop towards different sources of natural compounds, e.g. plant extracts, bacteria and derivates, and entomopathogenic fungi. Reported resistance include cases against pyrethrum, spinosyns, and *Bacillus thuringiensis* in insects^36,37^ and against polyoxin B in *Alternaria alternata*^38^. Although there are recorded cases, resistances against natural compounds are usually less pronounced than the ones against synthetic compounds, mostly due to the common presence of multiple modes of action in natural compounds^36,39^.

Although there are positive points in the performance of these natural products, the safety and success of their usage rely on factors such as type of compound, concentration, formulation, and application technology^29,40^. These are parameters extensively studied during the development of synthetic products. Since natural compounds used as plant protection agents follow the same statutory pathway as synthetic compounds, these parameters must also be studied for the development and registration of natural compounds.

The actual regulatory framework when it comes to natural compounds production in the United States (US), for example, is a responsibility of the Environmental Protection Agency (EPA), which register these compounds in the Biopesticides and Pollution Prevention Division (BPPD). There, applicants are benefited from tailored data requirements, reduced fees, and shorter time until product launch^41,42^. In the European Union (EU), the registration is done by the European Food Safety Authority (EFSA) and falls under Regulation (EC) No 1107/2009, together with other active substances^43^. Recently, data requirements for registration of natural compounds have been updated through Commission Regulation (EU) 2022/1439 in a way to align them to biological characteristics, with the intention of reducing the time for approval^44,45^.

These particularities show that the development of products based on natural compounds can be as extensive and laborious as for synthetic products. Previous research on PGAs, although their bioactivity has been tested against different pests and pathogens, focused on specific targets that are present in potato/Solanaceae cultivation system. Besides that, due to their toxicity to humans, a great part of the published literature related to PGAs has food safety as central topic.

By encompassing assays with microbial plant pathogens, a PPN, beneficial organisms, and a model plant, this study aims to broaden the effect and efficacy testing of α-solanine and α-chaconine in different biological groups. This, with the critical perspective of evaluating both the toxic power on target organisms, but most importantly, the not harmful characteristic towards non-target organisms. With our findings, we intend to increase the knowledge on the scope of control by PGAs and provide a new natural source to be used on the development of more sustainable crop protection products.

## Results

### The phytotoxic effect of PGAs causes impairment in seed germination and seedling development of *A. thaliana* Col-0

**Fig. 1:**
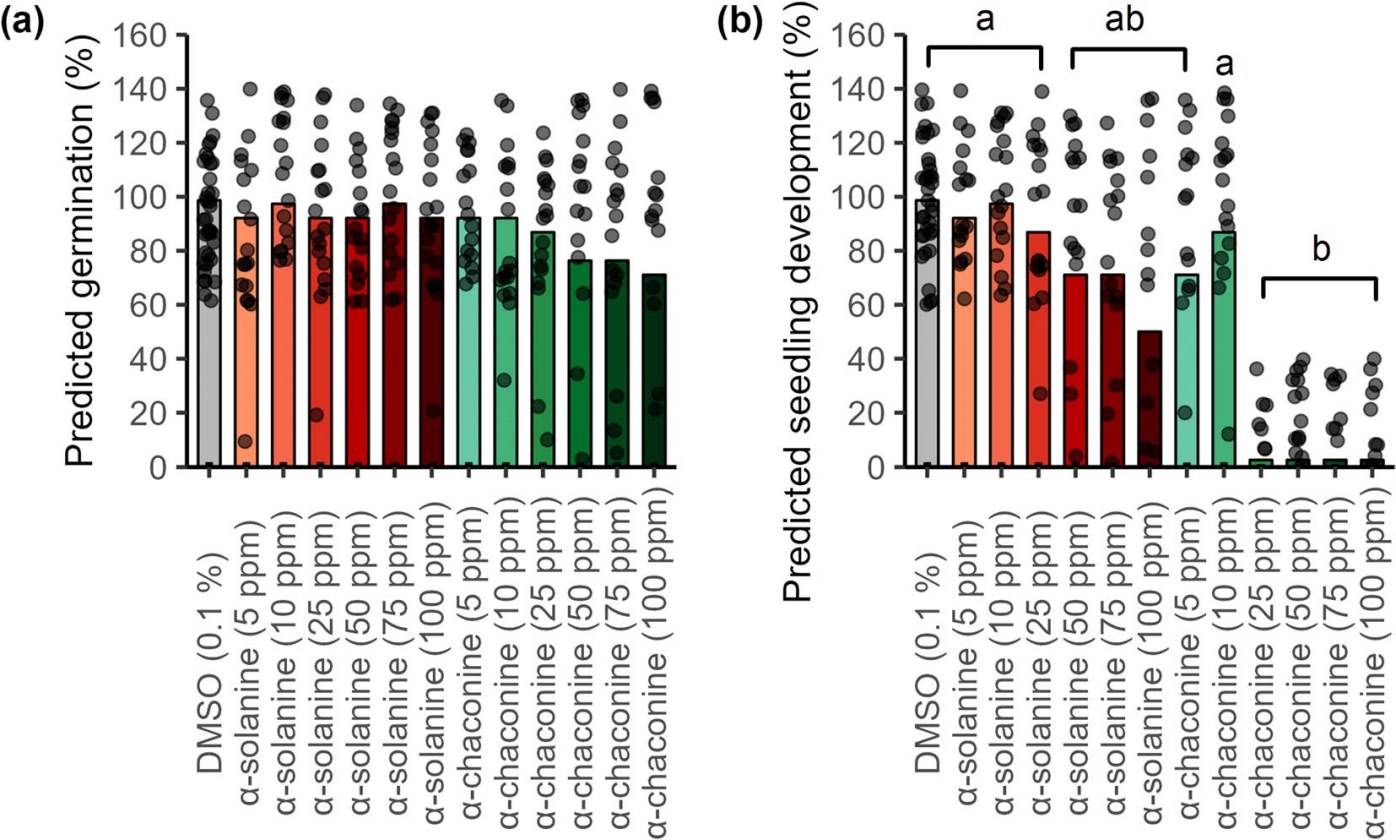
Effect of potato glycoalkaloids (PGAs) in the growth medium on the (**a**) germination and (**b**) seedling development of *Arabidopsis thaliana* Col-0 seedlings, compared to 0.1% DMSO (control). DMSO: dimethyl sulfoxide. Bars represent predicted germination/seedling development (%) estimated from binomial regression, with 95% confidence intervals. Jittered points show raw replicate data. Different letters indicate significant differences between treatments (Tukey’s HSD, *p* < 0.05). Data shown as raw data (*n* = 6 seeds per treatment, three biological replicates).

Plant protective compounds should not harm plants’ germination and growth. To investigate the phytotoxicity of the PGAs and the applicability of *A. thaliana* ecotype Col-0 in subsequent assays, a preliminary assay was conducted using six concentrations (5, 10, 25, 50, 75, and 100 ppm) of α-solanine and α-chaconine. In order to evaluate phytotoxic effects on seeds and seedlings, a scale was designed to assess developmental stages from “no germination” to “well-developed shoot and root system”. Fully developed seedlings were the ones phenotypically similar to control plants, with two big cotyledon leaves and a perfect root system with primary and secondary roots. Details of the different ranks are provided in the Methods section. Seed germination (Fig. 1a), meaning the rupture of the seed coat and radicle emergence, was not affected by either α-solanine (≤92.1% of germination success) or α-chaconine (≤71.05% of germination success), compared to 0.1% dimethyl sulfoxide (DMSO) (control). Critical phytotoxic effects in *A. thaliana* Col-0 seedling development were identified only for α-chaconine. This compound exhibited severe phytotoxicity already at 25 ppm (< 97% of development reduction) (Fig. 1b). This experiment was critical for selecting glycoalkaloid concentrations that avoid phenotypic interference in subsequent *A. thaliana* experiments.

### Harmful effects of α-chaconine on nematode viability, parasitism, and development

**Fig. 2:**
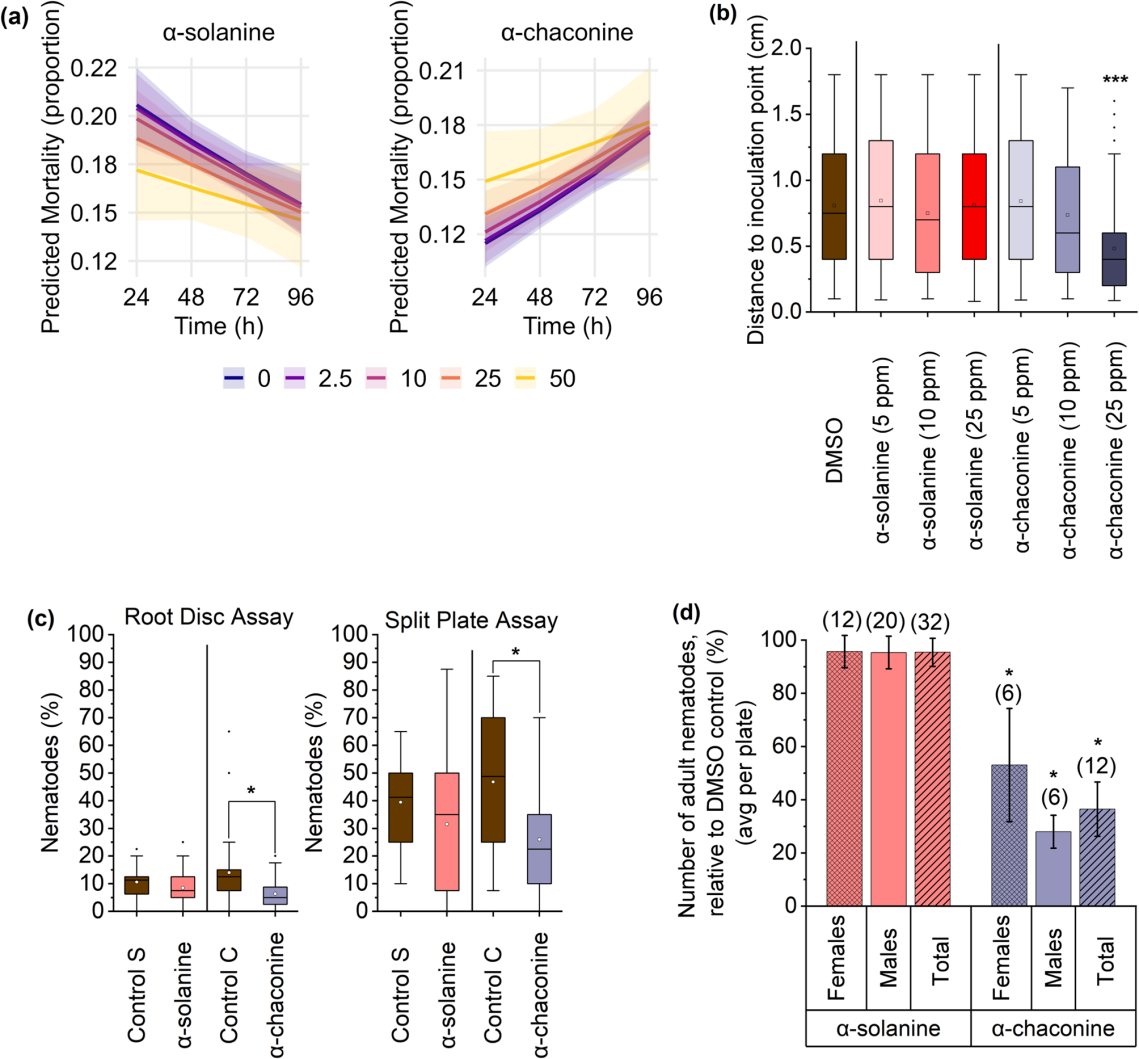
Effect of potato glycoalkaloids (PGAs) on the viability, mobility, attraction, and parasitism of *Heterodera schachtii*. (**a**) Infective juvenile (J2) mortality across PGA concentrations (0–50 ppm), analyzed by generalized linear model (GLM, binomial errors, logit link). Lines represent fitted probabilities; shaded areas include 95% CI. (**b**) J2 mobility after 90 min on media with different PGA concentrations, shown as distance from the inoculation point (boxplots, Kruskal–Wallis, Dunn’s post hoc test with Bonferroni correction, *p* < 0.0001). (**c**) J2 attraction after 24 h, expressed as the proportion of J2 at the PGA site relative to the total on the Petri dish, with (Root Disc Assay) or without (Split Plate Assay) *A. thaliana* Col-0 roots. Attraction is shown as boxplots (Root Disc: Mann–Whitney test, *p* < 0.05; Split Plate: t-test, *p* < 0.05). Control: 0.01% DMSO; PGA: 10 ppm. S: α-solanine; C: α-chaconine. (**d**) Number of adult nematodes per plate relative to the control (0.01% DMSO; PGAs: 10 ppm), with absolute numbers in parentheses (one-way ANOVA, Tukey’s HSD, *p* < 0.05). Data are mean ± SE unless shown as boxplots. Sample sizes: *n* = 12 (**a**), 360 (**b**), 36 (**c** Root Disc), 18 (**c** Split Plate, **d**); three independent biological replicates. Asterisks (*) indicate significant differences compared to control.

A series of assays was conducted to investigate the effects of α-solanine and α-chaconine on *H. schachtii*. The first assay assessed the direct impact on infective juveniles (J2) by incubating them in solutions containing various concentrations of α-solanine and α-chaconine (2.5, 5, 10, 25, and 50 ppm) for 96 h. Overall, mortality decreased over time regardless of the concentration (Fig. 2a), but none of the compounds significantly affected J2 viability in our experiment. No significant increase in J2 mortality was observed for either compound (Fig. 2a).

Next, the effect of both PGAs on J2 mobility was examined by placing J2 on agar supplemented with 5, 10, or 25 ppm of each compound, followed by measuring the distance J2 covered within a period of 90 min (Fig. 2b). While α-solanine did not affect J2 mobility, α-chaconine exhibited a concentration-dependent inhibition, with 25 ppm significantly impairing J2 movement. As revealed by visual observation, at 5 ppm α-chaconine, J2 moved freely to the edges of the Petri dish, whereas at 25 ppm, most J2 remained near the inoculation point in the center.

The third assay evaluated the effect of PGAs on nematode attraction using two setups: a “Root Disc” assay with the presence of *A. thaliana* roots, and a “Split Plate” assay without *A. thaliana* roots, in a dual-choice setting (10 ppm PGA and DMSO control) (Fig. 2c). The percentage of J2 present in control and PGA-supplemented agar discs or areas was compared to the total applied. Only α-chaconine significantly reduced J2 attraction: about 45% in the absence of roots and even 54.7% in the additional presence of roots compared to the respective control (0.01% DMSO). α-Solanine had no significant effect in either setup.

Since 10 ppm of either PGA did not affect plant root development or J2 fitness and mobility, this concentration was selected for an infection assay to assess their effects on nematode parasitism and development using *A. thaliana* Col-0 as host plant (Fig. 2d). α-Solanine did not affect nematode parasitism, whereas α-chaconine significantly reduced the number of adult nematodes per plant. Treated plants averaged 6 females and 6 males per plate, 46% and 72% fewer, respectively, than the control (0.01% DMSO). Female size and egg production per plant were not significantly affected (Supplementary Fig. S1 online).

### Glycoalkaloids induce a rapid and strong oxidative response in *A. thaliana* roots, with evidence for suppression of elicitor-induced ROS

**Fig. 3:**
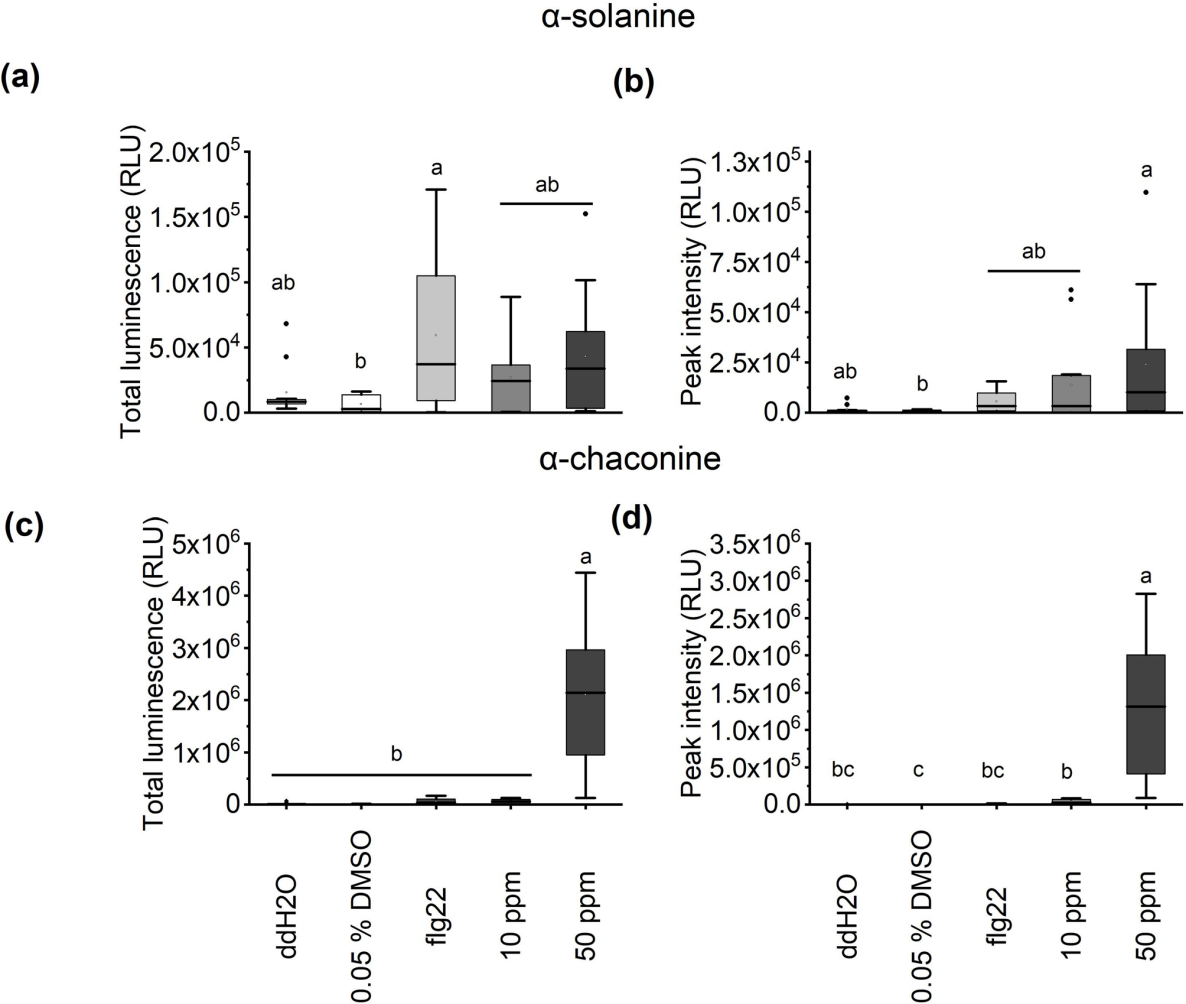
Reactive oxygen species (ROS) response of *Arabidopsis thaliana* Col-0 roots to α-solanine and α-chaconine. (**a**) Accumulated total ROS in relative light units (RLU) produced within the first 25 min after the addition of 10 or 50 ppm α-solanine. (**b**) Luminescence peak intensity after treatment with α-solanine. (**c**) Accumulated total ROS in relative light units (RLU) produced within the first 25 min after the addition of 10 or 50 ppm α-chaconine. (**d**) Luminescence peak intensity after treatment with α-chaconine. DMSO: dimethyl sulfoxide; ddH_2_O: double-distilled water. Letters indicate significant differences among the treatments for the parameters: total luminescence and peak intensity by Kruskal-Wallis-ANOVA and Dunn’s test with Bonferroni correction (*p* < 0.05). Boxplots show the median (horizontal line), mean (square), interquartile range (25th–75th percentiles, box), whiskers extending to 1.5 × IQR, and individual points outside this range are plotted as outliers (filled circles). *N* = 12 and three independent biological replicates.

**Fig. 4:**
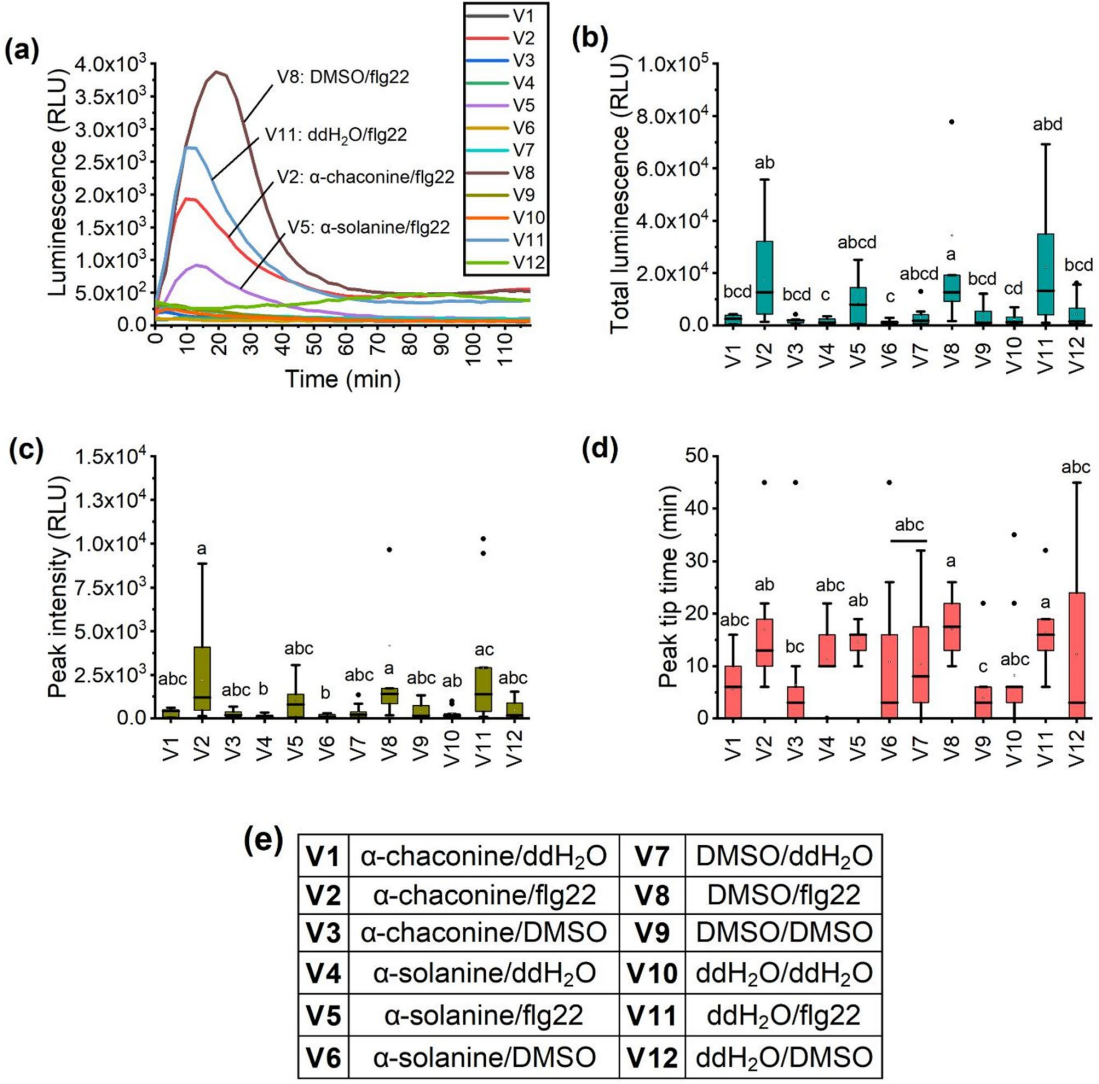
Reactive oxygen species (ROS) response of *Arabidopsis thaliana* Col-0 roots to flagellin 22 after pretreatment with 10 ppm α-chaconine or α-solanine. (**a**) Luminescence measured for 2 h in relative light units (RLU) after the addition of 50 µL of flagellin 22 (flg22, 1µM). (**b**) Accumulated total ROS produced within the first 45 min of the measurement time of the assay presented in Fig. 4a. (**c**) Luminescence peak intensity and (**d**) time of peak tip for the data presented in Fig. 4a. (**e**) Description of the treatments (pretreatment/trigger). DMSO: dimethyl sulfoxide; ddH_2_O: double-distilled water. Treatments (Pretreatment/Trigger). Letters indicate significant differences among the treatments for the parameters (**b**) total luminescence, (**c**) peak intensity, and (**d**) peak tip time by Kruskal-Wallis-ANOVA followed by Dunn’s test with Bonferroni correction (*p* < 0.05). These parameters were represented for the first 45 min. Boxplots show the median (horizontal line), mean (square), interquartile range (25th–75th percentiles, box), whiskers extending to 1.5 × IQR, and individual points outside this range are plotted as outliers (filled circles). *N* = 12 and three independent biological replicates. *N* = 10–12 and three independent biological replicates.

Considering the previous observations showing that PGAs can reduce nematode attraction to and infection of *A. thaliana* roots, we investigated their capability to elicit or modify ROS synthesis in the roots of 2-week-old *A. thaliana* Col-0 seedlings (Figs. 3 and 4). Exposure to either α-solanine or α-chaconine immediately resulted in a strong, but transient ROS burst in a concentration-dependent manner. For α-solanine, ROS levels reached 2.4 × 10^4^ RLU at 50 ppm and 1.3 × 10^4^ RLU at 10 ppm (Supplementary Fig. S2 online). α-Chaconine triggered a much stronger oxidative response: with 3.5 × 10^4^ RLU about 2.6 times higher at 10 ppm, and with ~ 1.3 × 10^6^ RLU about 55 times higher at 50 ppm (Supplementary Fig. S2 online). Following this, the ROS production fell below the level of the water control within only a few minutes during the α-solanine treatment at 50 ppm whereas the ROS level induced by the same concentration of α-chaconine lasted for about 30 min. Total accumulated luminescence was higher in the flg22 control, likely because the α-solanine response was too rapid to capture the full peak and declined more quickly (Fig. 3a, S2 online). Even though, the ROS peak induced by α-solanine exceeded that of flg22 (a 22-amino acid peptide originating from bacterial flagellin) by 8.2 × 10³ RLU at 10 ppm and 1.8 × 10^4^ RLU at 50 ppm (Fig. 3b). The 50 ppm α-chaconine treatment resulted in significantly higher total luminescence in the first 25 min compared to all other treatments (Fig. 3c). Both concentrations of α-chaconine induced an immediate ROS response, with peak times occurring earlier than the flg22 control (Fig. S2 online). flg22 was used as positive control in our experiments due to its well-described ability to act as ROS elicitor.

To determine whether PGAs alter the root ROS response to flg22, *A. thaliana* roots were pre-incubated overnight with 10 ppm α-solanine or α-chaconine, washed, and then treated with flg22. Figure 4a shows that pretreatment with either compound led to the same trend of lowering the flg22-induced ROS response, with α-solanine causing a slightly greater decline than α-chaconine. During the first 45 min, total luminescence and peak intensity were higher in negative controls (0.01% DMSO and ddH_2_O) than in PGA pretreatments (Fig. 4b, c).

### PGAs affect the development of fungi and oomycete

Five different fungal species and one oomycete were grown on agar supplemented with various PGA concentrations (15.13–250 ppm) and assessed when control wells reached at least 80% mycelial coverage. Table 1 summarizes mean mycelial growth inhibition (%) relative to the control (0.25% / 0.125% (*Rhizoctonia solani*) DMSO). Both PGAs reduced pathogen colony growth, but for the majority of pathogenic organisms, α-chaconine was more effective than α-solanine. For example, at 62.5 ppm, α-solanine inhibited *Fusarium graminearum* growth 4.35 times less than α-chaconine at the same concentration. *Leptosphaeria maculans* was the most sensitive, with 250 ppm α-chaconine causing 7% inhibition. Inhibition of *F. graminearum* and *R. solani* did not exceed 3%, even at the highest concentration tested. On the contrary, growth of the oomycete *Pythium ultimum* was only impaired by a treatment with α-solanine, whereas α-chaconine had no significant impact. Among beneficial fungi, *Trichoderma viride* was not affected by either compound, but the growth of *Drechslerella stenobrocha* was impaired at 62.5 ppm of αsolanine and at 31.25 ppm of α-chaconine.

**Table 1:** Relative growth inhibition (%) of plant-pathogenic and beneficial microorganisms on media supplemented with potato glycoalkaloids (PGAs) compared to growth on 0.25%/0.125% (*Rhizoctonia solani*) DMSO (control).

	Organism	Treatment (ppm)	Growth inhibition (%) (±SE)	
			α-solanine	α-chaconine
Plant-pathogenic	*Pythium ultimum*	31.25	3.87 (± 0.91)	1.65 (± 1.09)
		62.5	6.37 (± 1.85)*	0.82 (± 0.82)
		125	6.00 (± 0.68) *	1.66 (± 1.10)
		250	7.07 (± 2.47) *	0.77 (± 1.62)
	*Fusarium graminearum*	31.25	1.04 (± 1.04)	4.04 (± 2.84)
		62.5	2.49 (± 1.29)	10.83 (± 2.39) *
		125	3.94 (± 4.32)	12.24 (± 3.61) *
		250	16.21 (± 0.59) *	28.97 (± 3.51) *
	*Leptosphaeria maculans*	31.25	5.47 (± 4.87)	16.20 (± 13.45)
		62.5	14.40 (± 9.11)	11.82 (± 20.29)
		125	16.88 (± 12.87)	57.24 (± 9.40) *
		250	30.41 (± 16.84) *	78.41 (± 3.79) *
	*Rhizoctonia solani*	15.13	6.78 (± 1.26)	11.98 (± 2.01) *
		31.25	8.71 (± 3.30) *	12.91 (± 1.88) *
		62.5	11.04 (± 5.53) *	12.92 (± 3.26) *
		125	14.66 (± 4.47) *	23.50 (± 2.41) *
Plant beneficial	*Trichoderma viride*	31.25	− 2.79 (± 5.33)	− 0.44 (± 0.44)
		62.5	− 0.62 (± 4.72)	− 1.71 (± 3.99)
		125	− 0.46 (± 1.12)	− 1.50 (± 4.92)
		250	− 0.97 (± 2.81)	8.27 (± 11.22)
	*Drechslerella stenobrocha*	31.25	8.14 (± 3.59)	11.92 (± 1.92) *
		62.5	12.36 (± 5.69) *	13.80 (± 2.66) *
		125	12.88 (± 5.57) *	14.78 (± 1.31) *
		250	12.81 (± 5.89) *	19.59 (± 3.57) *

Experiment was carried out in different media, according to each microorganism: Potato Dextrose Agar (PDA) – *P. ultimum*, *F. graminearum*, *L. maculans*, *R. solani*; Malt Extract Agar (MEA) – *T. viride*. Asterisks (*) show significant differences compared to the control by one-way ANOVA followed by Tukey test (*p* < 0.05). Mean ± SE of three independent biological replicates (*n* = 12).

### Different impact of PGAs on bacterial growth

**Fig. 5:**
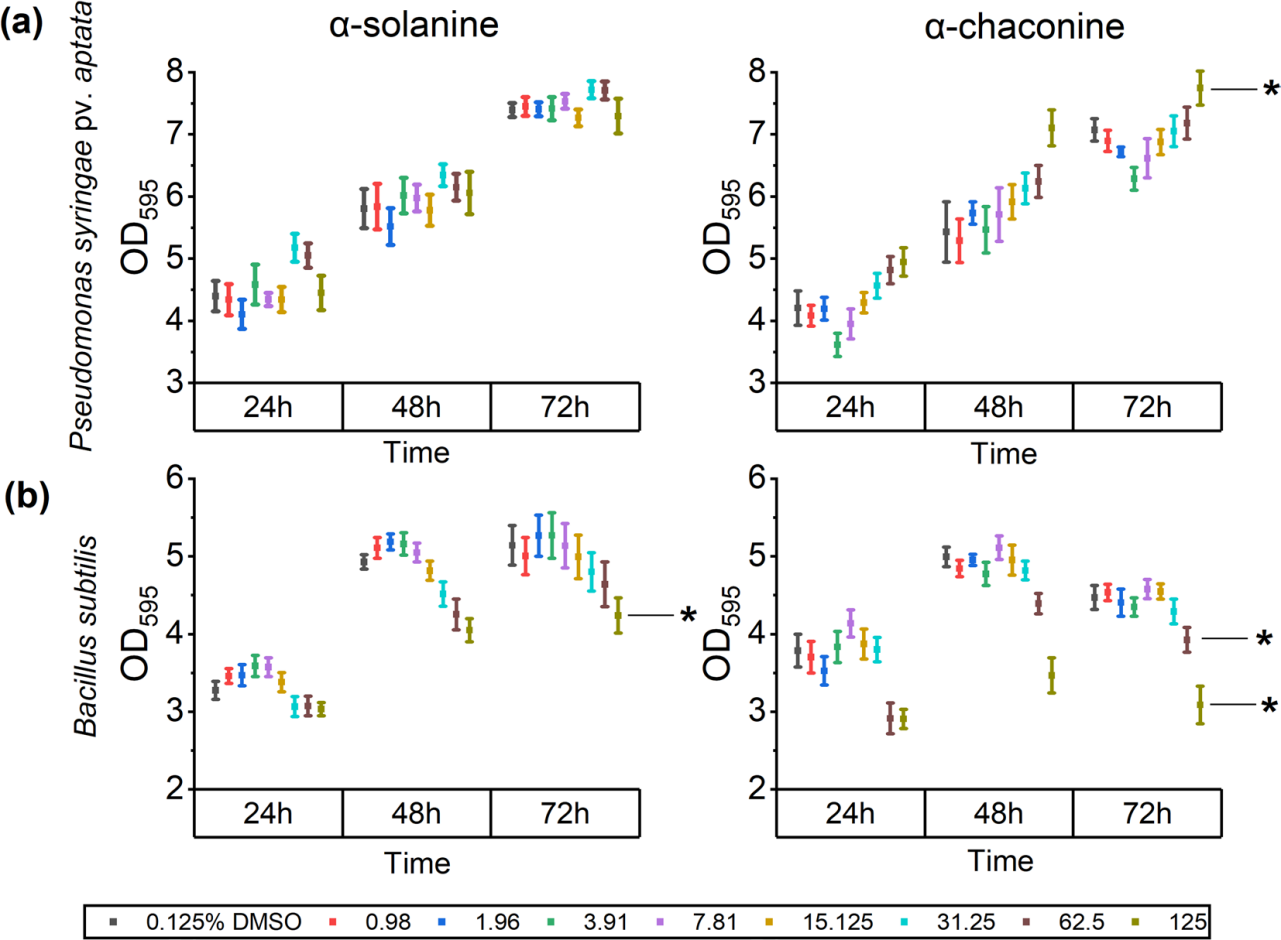
Growth of *Pseudomonas syringae* pv. *aptata* (**a**) and *Bacillus subtilis* (**b**) in liquid culture supplemented with PGAs at different concentrations (ppm). DMSO: dimethyl sulfoxide; OD_595_: optical density at 595 nm. Asterisks (*) show significant differences compared to the control by two-way ANOVA followed by Tukey test (*p* < 0.05). Mean ± SE of three independent biological replicates (*n* = 12).

PGAs had differing effects on bacterial growth, with α-solanine generally being less inhibitory. Suspensions of *Pseudomonas syringae* pv. *aptata* and *Bacillus subtilis* were incubated with various concentrations (0.98–125 ppm) of each glycoalkaloid for 72 h. *P. syringae* growth was improved by 18.5% compared to the control when in contact with α-chaconine at 125 ppm (Fig. 5a). For *B. subtilis*, higher concentrations of PGAs caused a reduction in growth (15.2% for α-solanine at 125 ppm and 15.3% and 28.6%,15.3% and 28.6% for α-chaconine at 62.5 and 125 ppm, respectively) (Fig. 5b), whereas lower concentration of α-solanine (up to 7.81 ppm) showed a positive impact on the growth.

## Discussion

This study examined the effects of α-solanine and α-chaconine on the growth and survival of various organisms, aiming to investigate the potential use of PGAs in developing novel plant protection products. To do so, the selection of target and non-target organisms was based on agricultural relevance, focusing on plant-parasitic cyst nematodes, key plant pathogens, and beneficial organisms. The tested organisms included a sugar beet pathogen and a parasite (*Pseudomonas syringae* pv. aptata, *Heterodera schachtii*, respectively), a crucifer pathogen (*Leptosphaeria maculans*), a cereal pathogen (*Fusarium graminearum*), and pathogens with a broader host range (*Pythium ultimum*,* Rhizoctonia solani*). Non-target organisms included established biological control agents (*Bacillus subtilis*,* Trichoderma viride*,* Drechslerella stenobrocha*) and the model plant *Arabidopsis thaliana* Col-0. In almost all tested groups, α-chaconine exhibited a stronger effect than α-solanine, causing greater impairment at the same or lower concentrations.

When testing the compounds on *A. thaliana* Col-0, α-solanine did not impair the seedling development even at 100 ppm. However, α-chaconine was already harmful at 25 ppm. Typical symptoms of alkaloid phytotoxicity include reduced seed germination, shortened hypocotyls, and inhibited root and shoot growth. In our observations, seedlings that could not develop further due to the presence of phytotoxic concentrations of α-solanine and α-chaconine demonstrated only the radicle emergence, or the formation of hypocotyl and small cotyledon leaves without any root system. López-Gonzalez et al.^46^ observed problems in *A. thaliana* root development already at low concentrations of the alkaloid norharmane (10 to 26 ppm). A negative effect on root development was recorded for mustard when in contact with α-solanine (43 ppm, 50.35% reduction)^47^ and for cucumber when in contact with α-solanine (87 ppm, 25.9% reduction) and α-chaconine (85 ppm, 35.2% reduction)^48^.

Regarding the plant-parasitic cyst nematode *H. schachtii* we observed that only α-chaconine negatively impacted nematode parasitism and development. This PGA reduced nematode mobility (25 ppm: 40%), attraction (10 ppm, with *A. thaliana* roots: 55%; without roots: 45%), and decreased the number of females and males (10 ppm: 46% and 72%, respectively). Similar findings were reported by Sivasankara Pillai and Dandurand^49^ for the potato cyst nematode *Globodera pallida*, where *Solanum* glycoalkaloids α-solamargine, α-solamarine, and the aglycone solasodine treatments reduced infection and reproduction, likely due to membrane disruption and inhibition of sensory perception. The activity of PGAs in microorganisms is primarily linked to their ability to alter membrane fluidity through interactions with sterol-containing membranes, ultimately resulting in membrane disruption^50^. According to the liposome model by Keukens^16^ the aglycone solanidine inserts into the lipid bilayer in a 1:1 ratio with membrane sterols, initially in a reversible manner. As the aglycone-to-sterol ratio increases, the sugar moieties of the glycoalkaloids interact irreversibly, forming a rigid matrix that ultimately leads to membrane lysis.

The difference in activity between α-solanine and α-chaconine is attributed to their sugar moieties^12–15^. Both PGAs share the same aglycone (solanidine) but differ in their carbohydrate side chains: α-solanine contains a solatriose group (glucose-galactose-rhamnose), while α-chaconine features a chacotriose group (rhamnose-glucose-rhamnose)^16^. The type and sequence of carbohydrate groups attached to the steroidal core directly influence glycoalkaloid activity^12^. Milner et al.^1^ reviewed several studies showing that chacotriose-containing glycoalkaloids generally have higher activity than those with solatriose groups. In pests and pathogens of *Solanum* species, these glycoalkaloids have physiological effects such as feeding deterrence and reduced reproduction rates. The bioactivity of rhamnose-carrying molecules is also observed in other compounds, such as glycolipids. Bredenbruch et al.^51^ showed that mono- and di-rhamnolipids (with one or two rhamnose molecules) effectively impair PPNs and, in the case of di-rhamnolipids, also enhance plant growth and trigger early plant defense responses.

The observations in the nematode infection assays prompted investigation into whether this effect might have been related to altered plant defense response triggered by PGAs. To address this, ROS accumulation in *A. thaliana* Col-0 roots was measured following exposure to α-solanine and α-chaconine. In general, our results showed an intense burst of ROS upon exposure to the compounds, followed by a rapid decline to nearly zero, remaining below those observed in the negative control for the remainder of the evaluation period. Considering the biphasic ROS dynamics described by Wi et al.^52^, it can be inferred that the intense exposure to the compounds likely triggered an acute oxidative response, leading to an activation of detoxifying mechanisms, and neutralization of ROS that endured throughout the recovery phase. Here we also observed differences among the tested concentrations, with ROS accumulation in treatments with 50 ppm of the compounds being higher than with 10 ppm. A concentration-dependent cytotoxic activity of PGAs, as described by Singh et al.^53^, may be attributed to the pronounced and transient nature of the ROS response.

Regarding a possible priming effect, neither α-solanine nor α-chaconine, when applied prior to the second trigger flg22, increased ROS response. These results counterpoint the ones reported by Bredenbruch et al.^51^ when working with di-rhamnolipids. In their study, the following ROS curve was altered, displaying a significantly more intense plant ROS response to flg22 after a treatment with dirhamnolipids. It was concluded that these molecules act as a priming stimulus that enables the plant to react stronger upon a second stimulus like a biotic or biotic-related stressor like flg22. In our case, a possible explanation is the sterol-binding nature of PGAs, which confer them a pore formation ability^16^. This, could disrupt *A. thaliana* root cell membranes by interacting with sitosterol, stigmasterol, and campesterol^54^.

In normal conditions where the cell membrane is not disrupted, but damage-associated molecular patterns (DAMPs) released from damaged or stressed neighboring cells are present, the expression of receptor such as FLS2 is upregulated and the pattern-triggered immunity (PTI) is activated^55^. Membrane disruption by pore-formating PGAs mislocalize receptors and co-receptors like FLS2 and BAK1, and the clustering or flg22-binding might be impaired, resulting in reduction and/or delay of ROS accumulation^56,57^. Furthermore, the plant becomes weak and not able to defend itself from external infections. This effect may influence the nematode infection, since these parasites require a healthy and non-stressed plant to successfully infect and develop^58,59^. While these observations clearly show that PGAs at a concentration of 10 ppm have no priming activity, further molecular-level studies are advised to better understand the precise role of glycoalkaloids in plant defense mechanisms.

Regarding the effect on microorganisms, PGAs have demonstrated promising antifungal properties. Recent research has explored their effectiveness in inhibiting fungal and oomycete growth, revealing that their impact varies depending on the pathogen species, concentration, and experimental conditions as it was also observed in this study. Li et al.^60^ showed that PGAs extracted from potato tubers’ green peel, prepared at a high concentration (5 g/mL PGAs crude extract, containing 4 mg of α-solanine and 7.29 mg of α-chaconine), significantly inhibited *Fusarium sulphureum*.

In tuber assays, the lesion growth rate in treated samples (with PGA supplementation) was lower than in control samples (without PGA supplementation). The results observed showed a growth rate that increased from 59.97% at 4 days post-inoculation (dpi) to 509.96% at 16 dpi for treated samples. Meanwhile, control samples presented an increase from 98.18% (4 dpi) to 795.09% (16 dpi). In in vitro conditions, the same PGA concentration reduced mycelial growth by about 88% compared to the control (only PDA). However, it is important to consider that the extracts were prepared in methanol, which may have contributed to the observed inhibition. Similarly, Pane et al.^61^ reported that potato leaf extracts from different cultivars negatively affected the growth of *R. solani*. For instance, the cultivar Luminella, with 492.4 ppm of α-solanine, reduced mycelial growth by over 60% Furthermore, treatments with Luminella extract also resulted in increased hyphal diameter (15.55 μm versus 11.77 μm in controls), indicating morphological changes induced by the extract. *R. solani* was also the target of our study, but growth inhibitions were not as expressive as in Pane et al.^61^. This can be attributed to the lower concentration to which this fungus was exposed (125 ppm), which brought a growth reduction of less than 16% in treatment with α-solanine and 24% in treatment with α-chaconine.

Considering similar concentrations to what was tested in the current study, Sánchez-Maldonado et al.^50^ demonstrated that *Alternaria alternata* and *Pyrenophora* sp. were more sensitive to α-chaconine at 170 and 200 ppm, respectively. Other fungi, such as *Aspergillus niger* and *Fusarium graminearum* were more resistant to the presence of α-chaconine and only showed impairment with concentrations above 800 ppm. *F. graminearum* was also the target of the current study but was not in contact with more than 250 ppm of PGAs. Positive results were observed for beneficial fungi where PGAs caused low to no impairment even at high concentrations. Pacifico et al.^62^ attribute this resistance to the enzymatic hydrolysis of PGAs into less toxic forms (e.g., solanidine, γ-solanine, and γ-chaconine). Other studies have shown that *Fusarium caeruleum* can hydrolyze α-solanine^63 ^and bacteria from the *Arthrobacter* genus can deglycosylate glycoalkaloids, using the resulting carbohydrates as a carbon source^64^. The highest efficacy recorded was against *Leptosphaeria maculans*, with growth inhibition of up to 79%. Currnt management of *L. maculans* relies on crop rotation and resistant varieties^65^. Although these results were obtained in vitro, they suggest strong potential for PGAs in controlling this pathogen, warranting further in vivo studies.

In the present study, *Pythium ultimum* (Order: Peronosporales) was the only target studied belonging to the oomycetes group, and our results showed that only α-solanine (62.5 ppm) inhibited its growth. The activity of this PGA was also previously tested for *Phytophthora infestans* (Order: Peronosporales)^19 ^and the authors also proved the effect of α-solanine (40.6 ppm) in reduced zoospore-mobility. When we compare the results between true fungi and oomycete, it is possible to notice some differences regarding the type and concentration of the tested PGA. One possible explanation for these differences may relate to membrane sterol composition. According to Gaulin et al.^66^, different types of sterol molecules are present in different kingdoms. While fungi contain ergosterol, oomycetes are most likely to contain lanosterol and/or fucosterol (Order: Saprolegniales). Some of the representants (Order: Peronosporales) are even unable to synthesize sterols and must scavenge from the host. Since the activity of α-solanine and α-chaconine have been previously related to sterol-binding, followed by pore-formation in the membrane^16 ^the absence of endogenous sterols may represent a critical factor influencing the effectiveness of PGAs.

In case of bacteria, *P. syringae* pv. *aptata* showed a small increase in growth when exposed to α-chaconine (125 ppm). One possible explanation is the nature of its outer membrane. As a Gram-negative bacterium, *P. syringae* pv. *aptata* possesses an outer membrane rich in lipopolysaccharides, which likely enhances its resistance to these compounds^67^. This structural barrier may reduce its susceptibility to glycoalkaloids. On the other hand, *B. subtilis*, which is a Gram-positive bacterium lacking the protective outer membrane^68 ^was negatively affected by α-chaconine (62.5 and 125 ppm), while α-solanine slightly reduced its growth in low concentrations. Other *Bacillus* species have also been reported to be susceptible to glycoalkaloids such as solamargine and solasonine, as well as *S. nigrum* extracts^67,69^. The growth improvement promoted by low concentrations allied with growth reduction at high concentrations observed here might suggest a biphasic pattern of toxicity within the framework of hormesis^70^. According to Calabrese and Mattson^71 ^hormesis is a resilience phenomenon present in biological systems (bacteria, plants, and humans), where low doses of toxic substances activate signaling of protective processes, e.g. induction of enzymes, upregulation of survival genes, activation of stress-response pathways, improving the overall organism. Additionally, certain bacteria, such as those from the *Arthrobacter*, *Serratia*, and *Alkalihalobacillus* genera, can metabolize glycoalkaloids, using the breakdown products for growth^64,72^. Within the *Pseudomonas* genus, *P. fluorescens* has been shown to detoxify PGAs^64 ^though the mechanisms in *P. syringae* remain largely unexplored.

Overall, these findings highlight the organism-specific effects of PGAs, with α-chaconine showing strong activity against key plant pathogens and parasites like *L. maculans* and *H. schachtii*, but also variable impacts on beneficial microbes. Although these results are indicative of how PGAs might impact different organisms, the method of exposure (where organisms were in permanent direct contact with the compounds in medium) is artificial and validation studies utilizing other application methods more common in plant protection practices (such as soil drench or spray) are needed. The positive results observed in this in vitro study underscore the need for further research to fully understand the in vivo effects and potential applications of PGAs as sustainable alternatives to synthetic plant protection products. Expanding studies to a broader range of organisms will be essential to bridge the gap between laboratory results and practical use in agriculture.

## Methods

### Chemicals

Reference standards of α-solanine and α-chaconine were purchased from PhytoLab GmbH & Co. KG (Vestenbergsgreuth, Germany) and dissolved in 100% DMSO upon arrival to an initial concentration of 100.000 ppm. Stock solutions of 500 ppm were prepared in double-distilled water (ddH_2_O) using an ultrasonic bath until the complete dissolution of the compounds. ddH_2_O was also used to dilute the solutions to working concentration ranges similar to the ones found in cultivated potato varieties (5–150 ppm)^6,73,74^. Concentration ranges differed according to the organism, their tolerance towards the presence of DMSO and experimental design. For instance, for different assays involving *A. thaliana* Col-0, we work with a range from 5 to 100 ppm; for *H. schachtii*, assays were on a range from 2.5 to 50 ppm; fungi and oomycete (with exception of *R. solani*) were tested on a range from 31.5 to 250 ppm (*R. solani*: from 15.125 to 125 ppm), and bacteria on a range from 0.98 to 125 ppm. DMSO was used as a control for all experiments, with a concentration range from 0.01 to 0.25% according to the assay.

### Pre-screening: effect of PGAs on plant development

#### Plant material

*A. thaliana* Col-0 seeds were used in all plant experiments. For that, the seeds were surface-sterilized (1% sodium hypochlorite for 5 min, 70% ethanol for 5 min, and rinsed three times in sterile ddH_2_O) and kept at 4 °C until usage.

#### Evaluation of PGAs’ effect on seed germination and seedling development

Since different assays demanded the use of *A. thaliana* Col-0 as a host, a pre-test to check the effect of α-solanine and α-chaconine on plant development was performed on a 96-well microplate. Seeds were sown in 100 µL (per well) of Knop agar modified from Sijmons et al.^75^ (2% sucrose, 1 mL L^− 1^ B5 vitamin) and supplemented with individual compounds at the final concentrations of 5, 10, 25, 50, 75, and 100 ppm. DMSO (0.1% was used as a control. Plates were incubated in a growth chamber at 25 °C with a 16 h light and 8 h dark cycle for 7 days. For evaluation, a scale with ranks from 0 to 4 was considered to evaluate the seedling’s phenotype (0: no germination; 1: seedling with germinated hypocotyl; 2: seedling with germinated hypocotyl and one or two small cotyledon leaves; 3: seedling with radicle, two big cotyledon leaves, and long primary root; 4: seedling fully developed with presence of two big cotyledon leaves, long root system with primary and secondary roots, phenotypically similar to control plants). Germination was considered when there was rupture of seed coat and seeds were recorded as 0 (no germinated) and 1 (germinated). Seedling development was considered a success (1) when, at the time of evaluation, seedlings were in ranks 3 and 4. Seedlings in lower ranks were considered failure (0). The experiment was repeated three times with six technical replicates each.

### Effect of PGAs on the cyst nematode *Heterodera schachtii*

#### Nematode inoculum preparation

The following setup was repeated for all the experiments described below: ~300 cysts of *H. schachtii* were harvested from a sterile mustard (*Sinapis alba* cv. Albatros) culture and transferred to a Baermann’s funnel, where cysts were soaked in 3 mM zinc chloride solution for seven days. Funnels were kept at 25 °C in the dark until J2 were harvested.

#### Evaluation of PGAs’ effect on nematode mortality

The direct effect of α-solanine and α-chaconine on *H. schachtii* larvae was evaluated on a microplate where 30–40 J2s were incubated in 200 µL of isolated compounds per well. The concentration ranged from 2.5, 5, 10, 25, to 50 ppm, and 0.05% DMSO was used as a control. Plates were incubated at 25 °C in the dark and evaluated on 24, 48, 72, and 96 h after inoculation. At the time of evaluation, 10 µL of 5 M NaOH was added to each well to induce movement in the live nematodes, making it easier to distinguish between live and dead nematodes^76^. The experiment was repeated three times with four technical replicates each.

#### Evaluation of PGAs’ effect on nematode mobility

To study the effect of α-solanine and α-chaconine on nematode mobility, modified Knop agar plates were supplemented with three different concentrations (5, 10, and 25 ppm) of the isolated compounds and 0.025% DMSO as a control. The experiment was performed based on the methodology previously described by Matera et al.^76^, with small modifications. 30 *H. schachtii* J2 were inoculated right in the center of the plate and incubated for 90 min. Posteriorly, the position of the nematodes was checked using a stereo microscope (Leica Microsystems, Wetzlar, Germany), marked down, and the distance was measured on ImageJ software^77^. The experiment was repeated three times with four technical replicates each. For statistics, it was considered the average of 30 J2 per plate.

#### Evaluation of PGAs’ effect on nematode attraction

Dual-choice assays were performed to study the effect of α-solanine and α-chaconine on nematode attraction. For the first experiment (Root Disc Assay), 10 ppm of each compound was used to supplement modified Knop agar plates, using 0.01% DMSO as a control. *A. thaliana* Col-0 seeds were sown onto these plates and incubated in a growth chamber at 25 °C with a 16 h light and 8 h dark cycle. Ten days after sowing, discs (~ 0.5 cm radius) containing roots of *A. thaliana* Col-0 were cut and transferred to water agar (8%). Each plate contained one disc with Knop agar supplemented with one of the PGAs (left) and another disc with the DMSO control (right). ~40 *H. schachtii* J2 were inoculated at the middle point between the two discs, and after 24 h, nematodes that were on the roots were recorded. For the second experiment (Split Plate Assay), plates were prepared with 50% of the area composed of modified Knop agar supplemented with 10 ppm of each isolated glycoalkaloid (left) and the other 50% with 0.01% DMSO (right). A disc (~ 0.5 cm radius) of normal modified Knop agar was placed in the intersection of these two halves, and ~ 40 J2s *H. schachtii* J2s were inoculated on top of this disc. The evaluation happened 24 h after inoculation by counting the number of nematodes located on the glycoalkaloid or control half. The experiment was repeated three times, with 12 technical replicates (with roots) and six technical replicates (without roots) each.

#### Evaluation of PGAs’ effect on nematode infection and development

To study the effect of α-solanine and α-chaconine on infection and development of *H. schachtii*, an assay was performed using *A. thaliana* Col-0 as the host plant. Ten days after sowing the seeds on modified Knop agar medium supplemented with 10 ppm of the isolated compounds (or 0.01% DMSO as a control), 70 *H. schachtii* J2s were inoculated on the roots. Plates were then incubated in a growth chamber at 25 °C with a 16 h light and 8 h dark cycle. Twelve days after inoculation, the number of adults (males and females) was recorded using a stereo microscope (Leica Microsystems, Wetzlar, Germany). The female size was evaluated 13 days after inoculation using Leica M165C Binocular and Leica Application Suite software (Leica Microsystems, Wetzlar, Germany). An additional evaluation of the number of eggs was performed 35 days after nematode inoculation. The experiment was repeated three times with six technical replicates each (six plates). Each replicate was composed of two *A. thaliana* Col-0 seedlings. Female size was assessed with a minimum of 30 females. For treatments containing fewer than 30 females, the sample size was set to the maximum number available for that treatment.

### Effect of PGAs on oxidative responses

#### Single trigger assay

To check the effect of α-solanine and α-chaconine on plant defense response, an analysis of oxidative burst when in contact with the PGAs was performed with root tissues of *A. thaliana* Col-0 based on the protocol described by Bredenbruch et al.^51^. For this, plants were sown onto modified Knop medium and placed in a growth chamber at 25 °C with a 16 h light and 8 h dark cycle for 14 days. To analyze the oxidative reaction, roots were harvested and added to 200 µL ddH_2_O overnight in the dark. The ddH_2_O was removed, and each well was filled with 35 µL of 1 mM Luminol (L-012: Pyrido [3,4-d] pyridine-1,4-dione, 8-amino-5-chloro-2,3-dihydro-7-phenyl) (Wako Chemicals GmbH, Germany), 15 µL of 20 µg/mL horseradish peroxidase. The elicitor solutions were added last: 50 µL of either a control (negative control: 0.05%DMSO and ddH_2_O, positive control: Flagellin-22 (flg22, 1 µM) (GenScript, USA)) or treatment (PGAs at 10 or 50 ppm) solution. After preparation, the luminescence was measured with a microplate reader (Infinite^®^200 PRO, Tecan Life Sciences Home, Germany). The experiment was repeated three times with four technical replicates each.

#### Dual trigger assay

A second assay was performed to analyze the effect of a pretreatment with PGAs on the ROS response to a subsequent stimulus from flg22. The setup was similar to the single trigger assay; however, in this case, the roots were incubated overnight in solutions with 10 ppm PGA or negative controls (0.01% DMSO and ddH_2_O). On the next day, plants received the same elicitation solution previously described, but the triggers were either flg22 or negative controls (Table 2). The experiment was repeated three times with four technical replicates each.

**Table 2:** Secondary trigger assay setup where *Arabidopsis Thaliana* Col-0 roots were pretreated with potato glycoalkaloids (PGAs) at 10 ppm and negative controls [dimethyl sulfoxide (DMSO) at 0.01% or double-distilled water (ddH_2_O)].

Variant	Pretreatment	Trigger
V1	α-chaconine	ddH_2_O
V2	α-chaconine	flg22
V3	α-chaconine	DMSO
V4	α-solanine	ddH_2_O
V5	α-solanine	flg22
V6	α-solanine	DMSO
V7	DMSO	ddH_2_O
V8	DMSO	flg22
V9	DMSO	DMSO
V10	ddH_2_O	ddH_2_O
V11	ddH_2_O	flg22
V12	ddH_2_O	DMSO

Triggering was performed with positive control flagellin-22 (flg22) or negative controls.

### Effect of PGAs on the growth of fungi and oomycete

Plant-pathogenic fungi, oomycete, and beneficial fungi used in this experiment are listed in Table 3. *T. viride*, *R. solani*, and *P. ultimum* were purchased from Leibniz-Institut DSMZ (Deutsche Sammlung von Mikroorganismen und Zellkulturen GmbH, Germany). Other organisms were obtained from laboratory collection. All colonies were frequently maintained on Potato Dextrose Agar (PDA) or Malt Extract Agar (MEA) (*T. viride*) and kept at 24 °C upon usage. The effect of α-solanine and α-chaconine was tested in a 24-well microplate, supplementing the respective media (300 µL) with a concentration range from 31.25 to 250 ppm (2-fold dilution) for all microorganisms, except *R. solani* (15.625 to 125 ppm). DMSO at 0.25 and 0.125% (*R. solani*) was used as a control. Plugs of mycelia (0.5 cm) were cut from maintenance cultures and placed in the middle of the well. The evaluation consisted of measurements of mycelial growth when the control replicates presented at least 80% of the media covered by mycelia. The percentage of inhibition was calculated relative to the respective control (Eq. 1). The experiment was repeated three times with four technical replicates each.

1 $$\:Growth\:inhibition\:\left(\%\right)=100-\left(\frac{Growth\:on\:media\:with\:glycoalkaloids\:\times\:100}{Growth\:on\:media\:with\:DMSO\:control}\right)$$

**Table 3:** List of plant pathogens and beneficial organisms used in the experiment.

Classification	Characteristic	Microorganism	Source
Oomycete	Plant-pathogen	*Pythium ultimum*	DSM (No. 62987)
Fungi	Plant-pathogen	*Leptosphaeria maculans*	DSM (No. 62910)
	Plant-pathogen	*Fusarium graminearum*	CBS (No. 5.15, Prof. Dr. Richard Sikora)
	Plant-pathogen	*Rhizoctonia solani*	DSM (No. 843)
	Beneficial	*Trichoderma viride*	DSM (No. 63065)
	Beneficial	*Drechslerella stenobrocha*	University of Bonn (Dr. Ulrike Steiner)

*DSM* German collection of microorganisms and cell cultures GmbH, Leibniz Institute, *CBS* Westerdijk Fungal Biodiversity Institute.

### Effect of PGAs on bacterial growth

The effect of α-solanine and α-chaconine was tested on the growth of two bacteria: *P. syringae* pv. *aptata* and *B. subtilis*. Working suspensions were prepared from overnight cultures to an initial optical density (OD_600_) of 0.1, using different liquid media for each bacterium (Luria Bertani Broth (LB) for *P. syringae* pv. *aptata* and Tryptic Soy Broth (TSB) for *B. subtilis*). 100 µL per well of bacterial suspension was added to a 96-well microplate containing solutions of isolated PGAs (100 µL per well) for a final concentration range between 0.98 and 125 ppm (2-fold dilutions). For all repetitions, 0.125% DMSO was used as a control. The plates were incubated on an orbital shaker (150 rpm) at 25 °C, and OD_595_ was measured at 24, 48, and 72 h using a microplate reader (Infinite^®^200 PRO, Tecan Life Sciences Home, Germany). The experiment was repeated three times with four technical replicates each.

### Statistics

Experiments were performed with at least three biological replicates, with technical replicates as previously described. Data were initially subjected to a normality test by Shapiro-Wilk (*p* < 0.05) and a Heteroskedasticity test by Breusch-Pagan test (*p* < 0.05) or overdispersion (ratio > 1), in case of binomial variables (seed germination and seedling development). Data that deviated from normal distribution and presented heteroskedasticity were tested by non-parametric tests, e.g. Kruskal-Wallis-ANOVA and Mann-Whitney Test. In this case, boxplots show the median, mean, interquartile range (25th–75th percentiles, box), whiskers extending to 1.5 × IQR, and individual points outside this range are plotted as outliers (filled circles). Parametric tests such as ANOVA (One-Way/Two-Way) and T-test were applied in normally distributed and homoscedastic data. In this case, means are displayed ± standard error (SE), and significances were tested by post-hoc tests accordingly. Seed germination and seedling development were analysed by binomial regression (0: no germinated/no developed: 1: germinated/developed). Nematode mortality was also analysed by binomial regression using counts of alive and dead nematodes in each timepoint. The analyses were performed in software Origin (Pro) (Version 2020)^78^ and R (Version 4.2.1)^79^.

## Supplementary Information

Below is the link to the electronic supplementary material.

Supplementary Material 1

## Data Availability

The experimental data that support the findings of this study are publicly available on PLANTdataHUB^80^ (https://git.nfdi4plants.org/usadellab/PGAsEffects).
