# Supplementary material for: In vitro effects of potato glycoalkaloids on plant-pathogens, beneficial microbes, and Arabidopsis thaliana
Source: Sci Rep. 2025 Sep 17;15:32584. doi: 10.1038/s41598-025-19637-9 (PMC12443959; doi:10.1038/s41598-025-19637-9)
Supplement: Supplementary file 1 — Supplementary Material 1 [file 41598_2025_19637_MOESM1_ESM.docx]

***In vitro* effects of potato glycoalkaloids on plant-pathogens, beneficial microbes, and *Arabidopsis thaliana***

Marília Bueno da Silva^1,2*^, Franziska Genzel^1,2^, Anika Wiese-Klinkenberg^1,2^, Sandra Bredenbruch^2,3^, Florian M. W. Grundler^2,3^, A. Sylvia S. Schleker^2,3,*^

^1^Institute of Bio- and Geosciences (IBG-4: Bioinformatics), CEPLAS, Forschungszentrum Jülich GmbH, 52425 Jülich, Germany

^2^Bioeconomy Science Center, BioSC, Forschungszentrum Jülich GmbH, 52425 Jülich, Germany

^3^Institute of Crop Science and Resource Conservation (INRES: Molecular Phytomedicine), University of Bonn, 53115 Bonn, Germany

* Corresponding author: ma.bueno.da.silva@fz-juelich.de, sylvia.schleker@uni-bonn.de

**Supplementary data**





**Fig. S1.** Effect of potato glycoalkaloids (PGAs) on **(a)** female size and **(b)** number of eggs/plant of *Heterodera schachtii*. No significant differences were observed for either parameter by ANOVA One-Way, p>0.05). Data are mean ± SE; n = 43-59 (a, two independent biological replicates), 36 (b, three independent biological replicates).





**Fig. S2.** Reactive oxygen species (ROS) response of *Arabidopsis thaliana* Col-0 roots to α-solanine.  **(a)** ROS response of root tissues was measured for 2 h in relative light units (RLU) after the addition of 10 or 50 ppm α-solanine. **(b)** ROS response of root tissues was measured for 2 h in relative light units (RLU) after the addition of 10 or 50 ppm α-chaconine. 50 µL of flagellin 22 (flg22, 1µM) served as positive control. DMSO: dimethyl sulfoxide; ddH_2_O: double-distilled water.
